# *Tyrophagus putrescentiae* (Sarcoptiformes: Acaridae) in the in vitro cultures of slime molds (Mycetozoa): accident, contamination, or interaction?

**DOI:** 10.1007/s10493-021-00608-4

**Published:** 2021-05-10

**Authors:** Dominika Michalczyk-Wetula, Monika Jakubowska, Magdalena Felska, Dariusz Skarżyński, Joanna Mąkol, Przemysław M. Płonka

**Affiliations:** 1 Department of Biophysics, Faculty of Biochemistry, Biophysics and Biotechnology, Jagiellonian University in Kraków, Gronostajowa 7, 30-387 Kraków, Poland; 2 Małopolska Centre of Biotechnology, Jagiellonian University in Kraków, Gronostajowa 7a, 30-387 Kraków, Poland; 3 Department of Invertebrate Systematics and Ecology, Wroclaw University of Environmental and Life Sciences, Kożuchowska 5b, 51-631 Wrocław, Poland; 4 Department of Invertebrate Biology, Evolution and Conservation, University of Wrocław, Przybyszewskiego 65, 51-148 Wrocław, Poland

**Keywords:** Storage mite, Interspecific interaction, Myxomycetes, *Fuligo septica*, *Physarum polycephalum*, *Didymium*

## Abstract

*Tyrophagus putrescentiae* (Schrank), commonly known as the cereal mite, cheese mite, or ham mite, is a cosmopolitan species reported from various environments in the wild, including soil, plant material and vertebrate nests. It has also been recognized as a common pest of food storages, mycological collections as well as plant and invertebrate laboratory cultures. Laboratory observations indicate that *T. putrescentiae* feeds on a large range of dermatophytes, yeasts and molds. We have observed the interspecific relation between this mite and several species of true slime molds (Mycetozoa) under laboratory conditions, which confirms the very broad spectrum of feeding habits of *T. putrescentiae*. Mycetozoans were grown in semi-sterile in vitro cultures and fed with oat flour or oat flakes. *Tyrophagus putrescentiae* displayed affinity to all macroscopically identifiable stages of the life cycle of *Fuligo septica* (L.) F.H. Wigg, *Physarum polycephalum* Schwein and the *Didymium* sp. complex [*Didymium iridis* (Ditmar) Fr., *Didymium nigripes* (Link) Fr. and *Didymium bahiense* Gottsb.]: live, decaying or dead plasmodia, sporangia, aethalia, spores and sclerotia. The relation carrying symptoms of various types of interspecific interaction, is hypothesized to form an evolutionarily young phenomenon, which not only identifies a new aspect of mycetozoal biology, but also presents the cereal mite as a species of high adaptive potential.

**Supplementary Information:**

The online version contains supplementary material available at 10.1007/s10493-021-00608-4.

## Introduction

Slime molds (Mycetozoa, called also Myxomycetes, Myxogastria, Myxomycota*,* Eumycetozoa) are simple but unusual eukaryotes. Their plasmodial structure and ameboid-type migration both justify their classification as gigantic microorganisms. Slime molds reveal some features of fungi (lack of autotrophy, production of sporangia in fruit bodies); however, many more characteristics of slime molds are shared with protozoans (Hamilton 2001; Espinosa and Paz-Y-Miño-C 2014). Because of displaying some intermediate traits, in addition to several primitive features, such as lack of histological organization of the body, large size and relatively easy cultivation under laboratory conditions, they have become macroscopic models of the eukaryotic cell and interesting alternate organisms in biological and biomedical research. Their popularity in interdisciplinary studies, such as biocomputing (Adamatzky 2010) or astrobiology (Díez et al. 2020), has increased rapidly.

A detailed description of the slime mold life cycle may be found in numerous handbooks and papers (e.g., Stephenson and Stempen 1994; Díez et al. 2020). Typically, their life cycle starts in sporangia with haploid spores surrounded by a cell wall. After germination, the haploid flagellated forms conjugate and, after a disposition of the flagella, the diploidal ameboid grows to the main vegetative form of the life cycle, the plasmodium. The spores may also be generated by apogamy and remain diploid, which makes the taxonomy of slime molds particularly challenging (Clark and Mires 1999; ElHage et al. 2000; Clark et al. 2001, 2004). The main plasmodial type, the so called phaneroplasmodium, is often pigmented and may reach the macroscopic size scale (up to tens or hundreds cm^2^; Stephenson and Stempen 1994; Płonka and Rakoczy 1997; Adamatzky 2010). The plasmodium preserves its ability to migrate actively. Under influence of starvation, drought, light, and other stimuli, the plasmodium creates a fruiting body, which may form a simple stalked or sessile sporangium, or a complex aethalium. Under adverse circumstances, the plasmodium may transform into a dormant state—usually the sclerotium that preserves its ability to regenerate into the active plasmodium under normal conditions (Krzywda et al. 2008).

Slime molds are heterotrophic, saprophytic organisms. In the laboratory they may grow on organic substrates (agar, gelatin, lignin, fluid media) absorbing nutrients from the feeder, but in their natural environment slime molds have developed various types of interactions with other organisms. At large, they feed on bacteria and other microorganisms, including fragments of other slime molds which they can absorb and digest (Madelin 1984; Stephenson 1989; Stephenson and Stempen 1994; Adamatzky 2010; Stephenson and Schnittler 2016). Some slime molds are parasitic. A known example, *Licea parasitica* (Zukal) G.W. Martin, associated with several species of lichens, seems to be a facultative lychenicolous mycetozoan of still unidentified ecological status (Kocourková 1999). Stephenson and Schnittler (2016) report some slime molds, including *Fuligo septica* (L.) F.H. Wigg, as capable of enzyme excretion to the substrate and extracellular digestion of fruiting bodies (and likely mycelia) of fungi. As commensals, slime molds can also compete with other organisms, e.g., strawberries (Arthaud and Lafon 1972), plant sprouts in hydroponic cultures (Michalczyk et al. 2011) or with cultivated mushrooms (Rakoczy and Szymański 1985; Chung et al. 1998).

Because their plasmodium (large amount of cytoplasm) represents a valuable source of nutrients, slime molds may fall prey to other organisms. It has been documented that slugs may feed on live plasmodia (Keller and Snell 2002; Stephenson and Schnittler 2016), and there is also quite a substantial literature on myxomyceticolous insects: beetles and flies (Stephenson and Stempen 1994; Stephenson and Schnittler 2016; Sugiura et al. 2019). These animals should be therefore called ‘insidious predators’ (Zelmer 1998). As production of spores may be attributed to primitive plants, algae or fungi, this type of interaction may also be called ‘grazing’ (Weiner 2003). However, reports on slime molds as the hosts for parasites are still infrequent, similar to those on the parasitic slime molds.

In our laboratory, on certain occasions, we have observed massive infestations of *Tyrophagus putrescentiae* in slime mold cultures. *Tyrophagus putrescentiae* is commonly referred to as the mold mite, cheese mite, or ham mite (Hughes 1961; Amoah 2016). It is a common pest of stored products, especially products with high fat and protein contents, such as whole wheat flour, soy flour, cheese, rye bread, white bread, herring meal, bacon, dry milk and various seeds (Hughes 1961; Duek et al. 2001). It has been reported to be associated with over 140 commodities (Hagstrum et al. 2013), including dried fruits, spices, cultured cheese, and other high-value foods (Rentfrow et al. 2006; Amoah 2016). Indeed, Keller and Smith (1978) and Smith and Keller (1978) reported an acarid mite, *T. putrescentiae*, feeding on a phaneroplasmodium. Observations of the interaction between the mite and an unidentified slime mold were made on live tree bark under laboratory conditions. They were followed by monitoring of this interaction in the in vitro agar cultures of slime molds of *Stemonitis flavogenita* Jahn., and *Didymium* sp. The authors acknowledged Guilford S. Ide (Curator, Acarology Laboratory, Ohio State University, USA), who identified the mite; however, in their short article, they did not include a list of character states that formed the basis for species identification.

Keller and Smith (1978) also observed that mites can positively interact with *Didymium* sp. by spreading its spores through the digestive system. In the present study, we expand these observations by: (1) confirming that the mite species which lives together with the slime molds is *T. putrescentiae*—we provide the set of character states which constituted the background for species identification; (2) proving that the mite can be found in cultures of various species of unrelated Myxomycetes, *F. septica*, various members of *Didymium* sp. and *Physarum polycephalum* Schwein, famous for its application in the field of biocomputing (Adamatzky 2010); and (3) revealing that the mites exhibit affinity to various stages of the slime mold life cycle (plasmodium, spores, sporangia and sclerotia). Finally, we discuss the nature of the ecological interaction between the mites and the slime molds.

## Material and methods

**Fig. 1:**
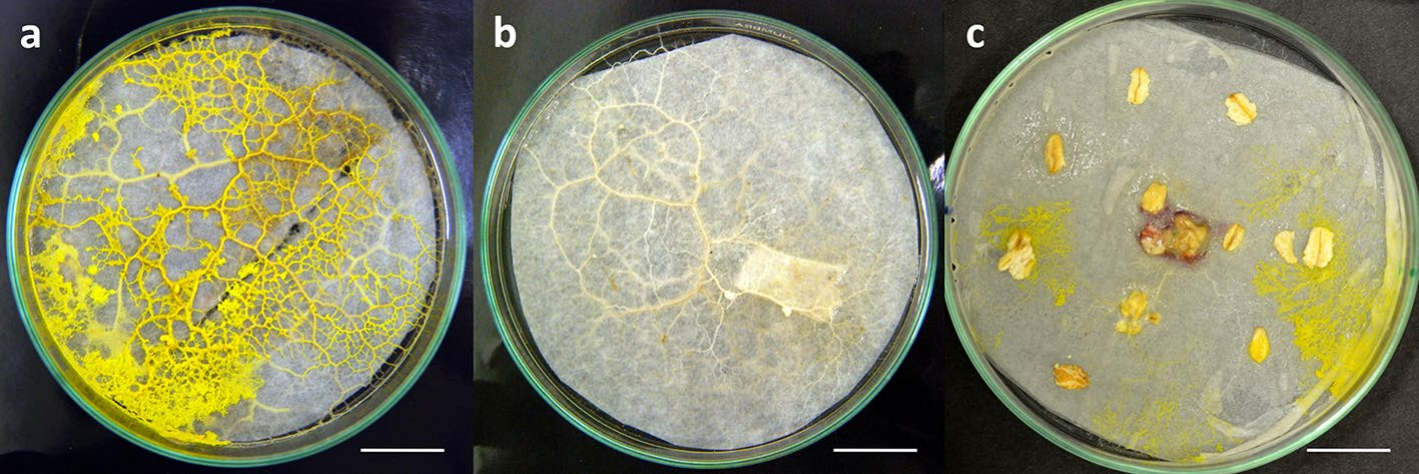
In vitro cultures of *Fuligo septica* (**a**), *Didymium* sp. (**b**) and *Physarum polycephalum* (**c**). All cultures on Petri dishes with filter paper and oat flour/flakes. Scale bar: 2 cm

Slime molds were cultured as described previously (Krzywda et al. 2008; Adamatzky 2010; Michalczyk et al. 2011) at room temperature (19–21 °C) in the dark, in glass Petri dishes (9 and 11 cm diameter), or in 5.5 cm diameter plastic tissue culture dishes (TPP Techno Plastic Products, Trasadingen, Switzerland). Humidity in the laboratory was low, ca. 30–35%, but within the covered Petri dishes it reached up to 84 ± 9% (mean ± SD; DM-9213 thermo-hygrometer, ATM, Hongkong, China). As *F. septica* is a unique agarolytic eukaryote (Murugan et al. 1996) which causes difficulties in maintaining it on solid agar (Haskins and Wrigley de Basanta 2008), the slime molds were grown on medium density Whatman® filter paper (Sigma-Aldrich, St Louis, MO, USA). They were fed with oat flour or oat flakes (some cultures of *P. polycephalum*) stored at −80 °C, and watered ad libitum with sterile water (Fig. 1). The cultures were serially passaged ca. 2–4 weeks under sterile conditions; however, we did not sterilize the flour/flakes. After passaging, the old cultures were maintained longer, and sometimes passaged a second or even third time. The mites were found in the old slime mold cultures, wherein they appeared most probably as contaminants of the oat flour/flakes.

The mites were preserved in 90% ethyl alcohol, mounted on microscopic slides in Swan’s fluid (Swan 1936), examined under Nikon Eclipse 50i microscope (Nikon Instruments, Tokyo, Japan) and identified based on the key provided by Solarz (2012). Photographs of *T. putrescentiae* were taken using the Nikon Eclipse 50i microscope coupled with a Toshiba 1080i camera system. The vouchers are deposited at the Department of Invertebrate Systematics and Ecology, Wrocław University of Environmental and Life Sciences, Poland.

Photographs of slime mold cultures were taken using a Nikon D7000 camera equipped with Nikon Nikkor Micro AF 60 mm 1:2.8 lenses (Nikon, Tokyo, Japan), and the video sequences were recorded using the same camera assembled to a Möller-Weder operating microscope (Möller-Wedel, Wedel, Germany). The sequences were edited using freeware OpenShot Video Editor v.2.4.4. (OpenShot Studios, Rockwall, TX, USA).

Density of mites in the culture dishes was estimated on the base of photographs and magnification (expressed as scale bars), and was only roughly determined, because of a very changeable occurrence of the mites (see, e.g., Smrž and Čatská 1987).

## Results and discussion

### Identity of the myxomyceticolous mite

**Fig. 2:**
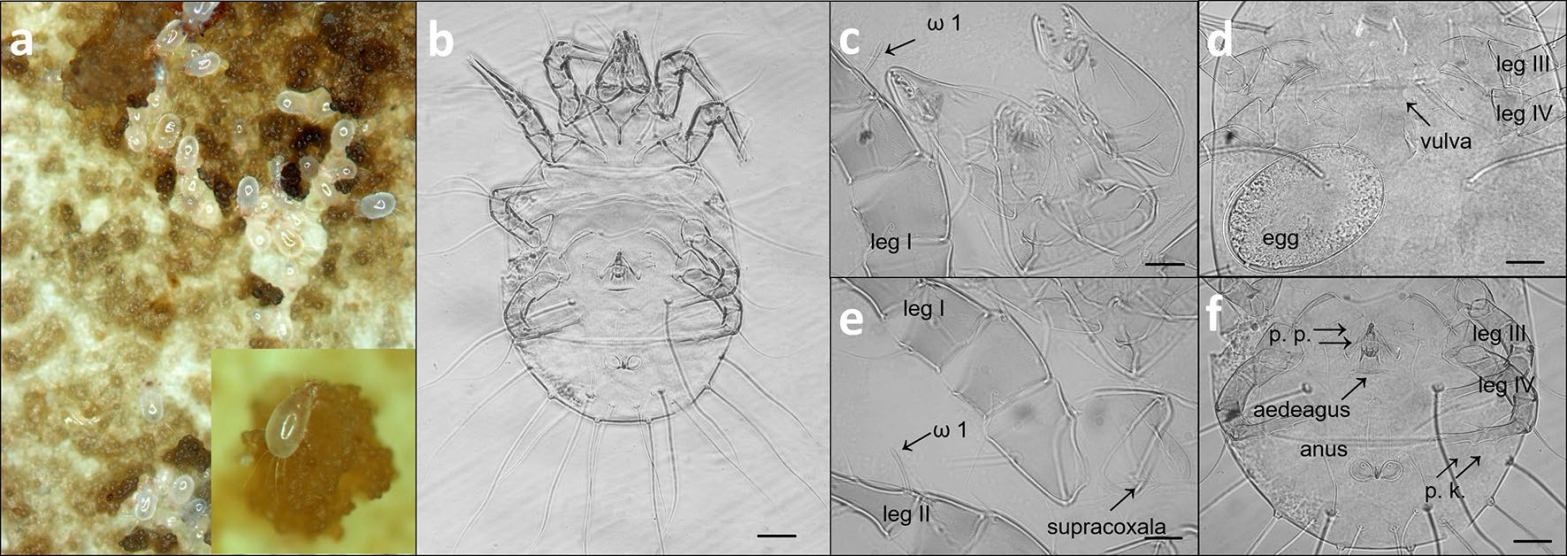
*Tyrophagus putrescentiae*. **a** Macrophotography of specimens in a culture, not to scale. **b** Male, ventral view. **c** Gnathosoma and leg I (part), dorsal view, ω 1 = solenidion 1 on tarsus I. **d** Female—hysterosoma, ventral view. **e** Gnathosoma (part) and legs I, II (part), dorsal view, ω 1 = solenidion 1 on tarsus II. **f** Male—opisthosoma and hind legs, ventral view. p. p., sex suckers; p. k., copulatory suction cups on tarsus IV. Scale bars: 45 µm (**b**), 13 µm (**c**, **e**), 28 µm (**d**, **f**)

The mites interfering with the slime molds represent *T. putrescentiae* (Sarcoptiformes: Acaridae). According to the data provided by Solarz (2012), the species can be distinguished based on the following characters: anterior margins of propodosomal shield with pigmented spots (corneae); seta *d 1* more than twice as long as *c 1*; seta *d 2* only slightly longer than *c 1*; seta *e 2* very long, similar in length to *h 2*; supracoxal seta (*sc x*) expanded at base, with 5–6 fairly long pectinations; distal 2/3 of solenidion ω 1 widened, terminated in a distinctly pointed tip. The diagnostic characters used to identify the species and to distinguish it from relatives are presented in Fig. 2—these are: seta *sc x* relatively long, either slightly or markedly widened in basal half, with long pectinations; tarsi I and II with solenidion ω 1 terminating in a distinctly expanded tip (distal 2/3 of solenidion ω 1 on tarsus I widened); S-shaped aedeagus in males, with two deep curves—one at the base, the other one in the apical third; tarsal (copulatory) suckers on Ta IV.

### Affinity to various species of Mycetozoa in all stages of their life cycle

**Fig. 3:**
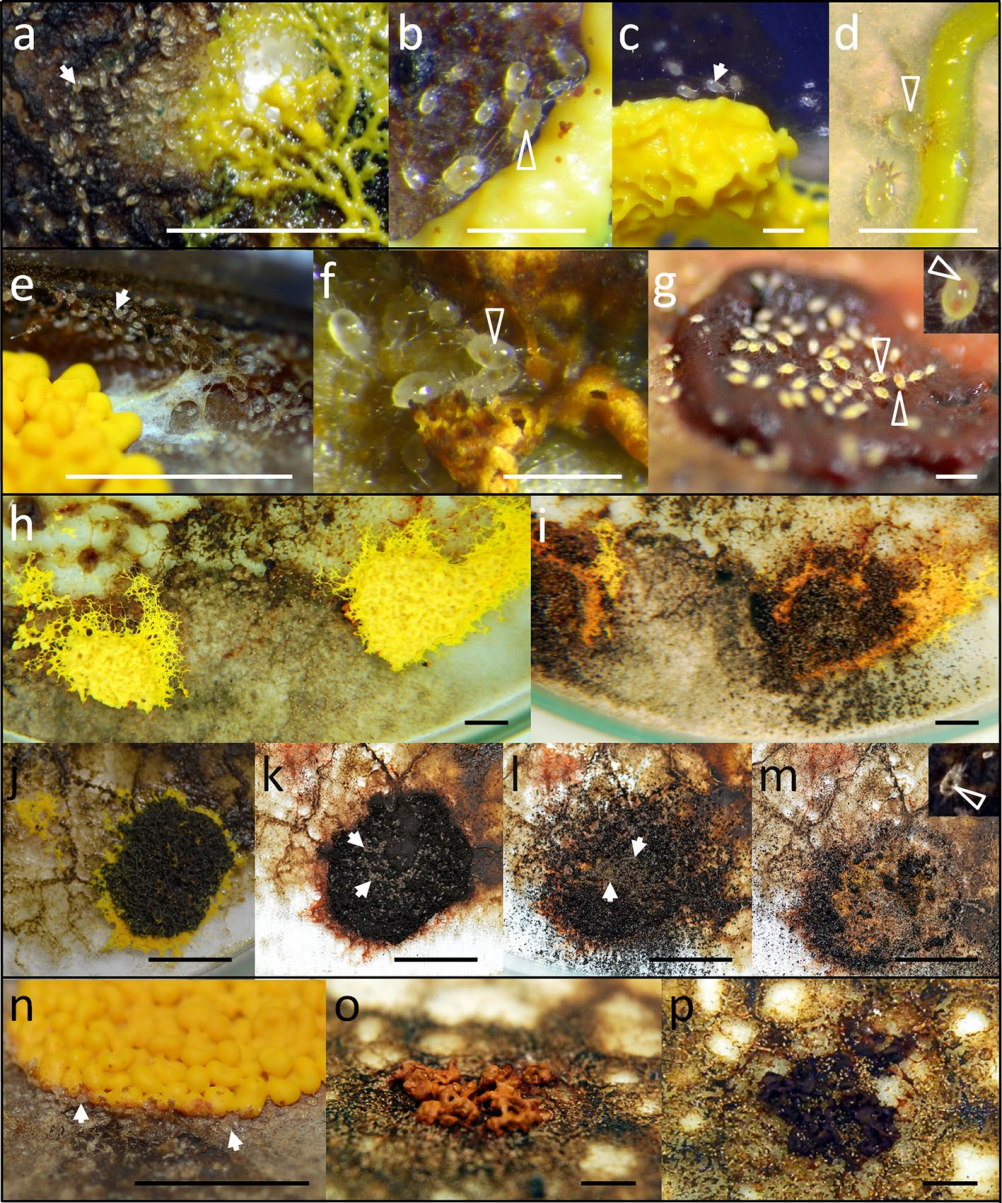
*Tyrophagus putrescentiae* (white arrows, when necessary) on various forms of *Fuligo septica*: on live (**a**–**d**) and dead (**e**–**g**) plasmodium (**e**—on the slimy track left by a plasmodium) of *F. septica*. Subsequent stages of two examples of the *F. septica* aethalium destruction after 8 days (**h**, **i**) and after 10 (**k**), 13 (**l**) and 17 (**m**) days following the onset of observation (**j**). *Tyrophagus putrescentiae* on a forming sclerotium of *F. septica* (**n**) and example of destruction of *F. septica* sclerotium within 24 h (**o**, **p**). White empty arrowheads indicate mites with their digestive tracts filled with various food items: yellow—live plasmodium (**b**, **d**), orange—dead plasmodium (**f**, **g**, **g**-inset), black—spores (**m**-inset). Scale bars: 0.5 cm (**a**, **e**, **h**–**p**), 1 mm (**b**–**d**, **f**, **g**)

**Fig. 4:**
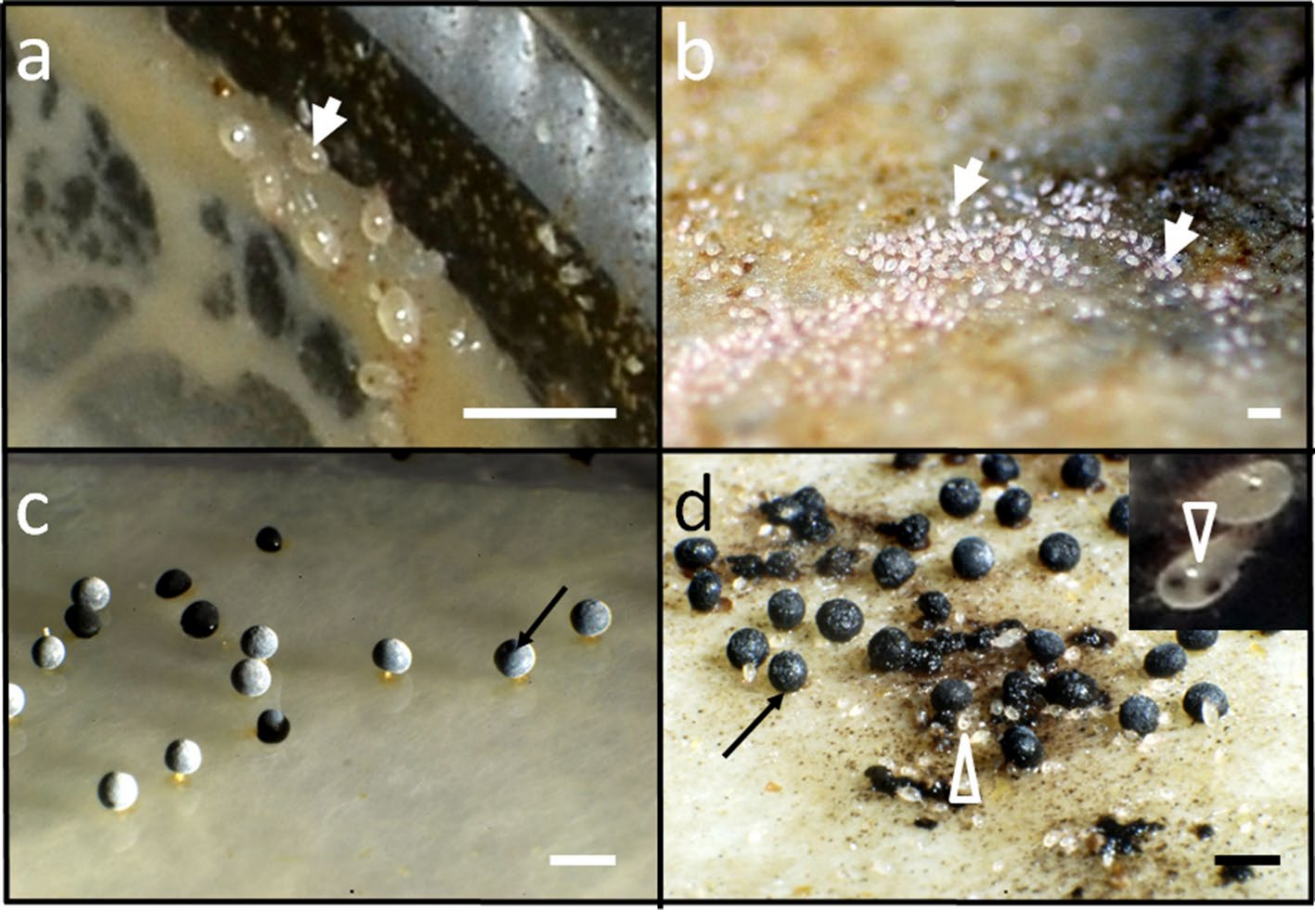
*Tyrophagus putrescentiae* on live (**a**) and dead (**b**) plasmodium of *Didymium* sp. Sporangium (black arrows) of *Didymium* sp. before (**c**) and after invasion of *T*. *putrescentiae* (**d**). White arrows and white empty arrowheads—as in Fig. 3. Scale bars: 1 mm

**Fig. 5:**
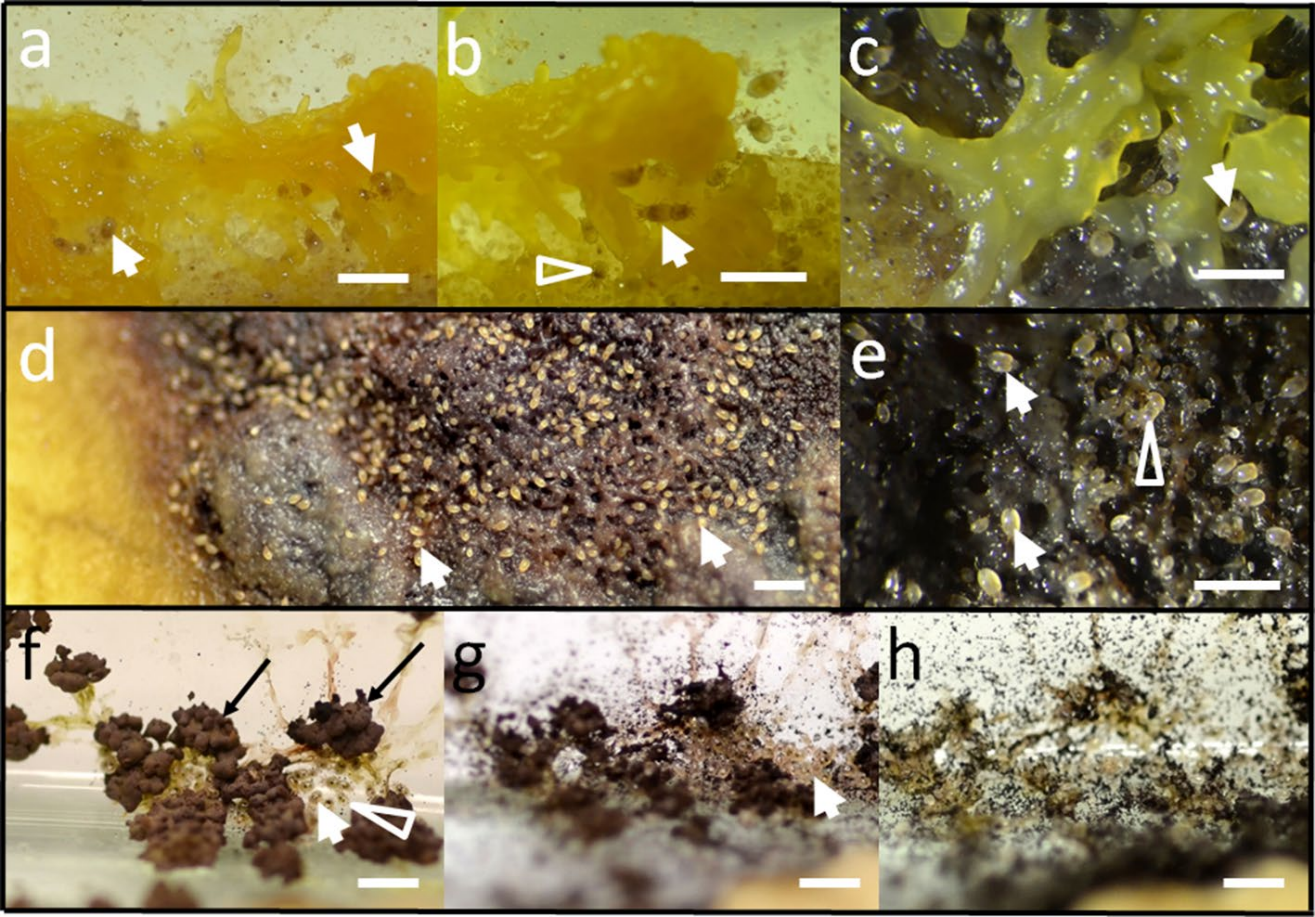
*Tyrophagus putrescentiae* on various forms of *Physarum polycephalum*: on live (white arrows) (**a**–**c**) and dead (**d**, **e**) plasmodium*.* Subsequent stages of the *P. polycephalum* sporangium destruction after 5 (**g**) and 8 (**h**) days after the initial observation (**f**). White and black arrows and white empty arrowheads—as in Figs. 3 and 4. Scale bars: 1 mm

The mites have been observed to feed on all macroscopic forms which develop in the life cycle of *F. septica*, *P. polycephalum*, and of several members of *Didymium* sp., earlier reported by Michalczyk et al. (2011) as a species complex, which reveals features of *Didymium iridis* (Ditmar) Fr., *Didymium nigripes* (Link) Fr. and *Didymium bahiense* Gottsb. (Stephenson 2003). It is important to note that these mycetozoans represent different family-level taxa. Whereas all belong to the order Physarales, *P. polycephalum* and *F. septica* represent family Physaraceae, and *Didymium* sp. is a member of Didymiaceae (Stephenson and Stempen 1994; Drozdowicz et al. 2003). The mites not only fed on plasmodia, but they also inhabited them (Online Resources 1) and make use of every identified stage of the slime mold life cycle. This included: live plasmodium (Figs. 3a–d, 4a, 5a–c, Online Resources 1), dead and decomposed plasmodium (Figs. 3e–g, 4b, 5d, e, Online Resources 2), aethalium (Fig. 3h–m, Online Resources 3) and sporangium (Figs. 4c, d, 5f–h), spores (Figs. 3i–m, 4d, 5f–h), and sclerotium (Fig. 3n–p) (Krzywda et al. 2008). We observed the same affinity as to *F. septica* (Fig. 3), also to *Didymium* sp. and *P. polycephalum* (Figs. 4, 5) in relation to the stages which we were able to follow under the in vitro conditions.

According to our observations the mites differed in their preferences to particular form of the slime mold material. Such preferences could be particularly well discernible when there were two or three forms of slime molds in a Petri dish. The mites concentrated around a particular form and then, after ingesting it, they moved to the other one. For example, if there was a dead fragment of plasmodium, besides a live one, the mites concentrated on the dead part first (e.g., Fig. 3e–g), and then moved to the live one. Thus, the following sequence of the preferences was established: dead or decaying plasmodium > spores/sporangia/aethalia > live plasmodium > sclerotia.

### Features of various types of ecological interactions

**Fig. 6:**
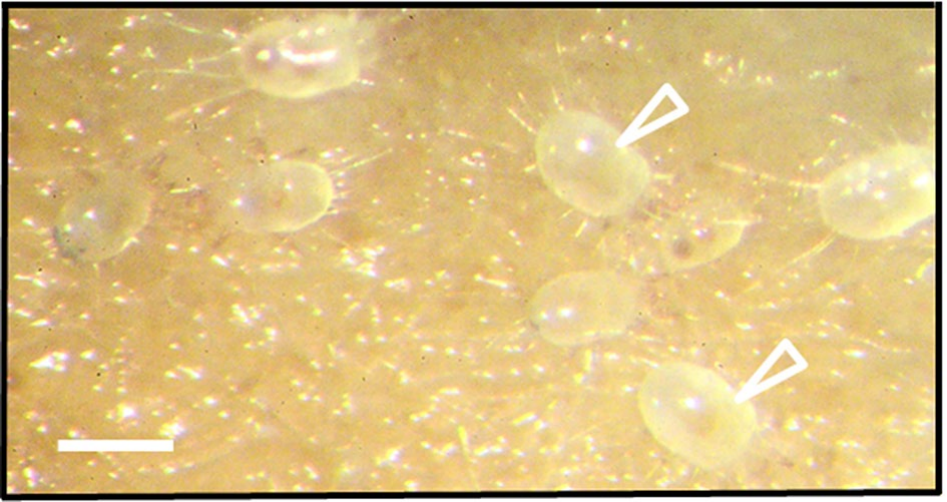
*Tyrophagus putrescentiae* in control cultures in Petri dishes with filter paper and oat flour but without slime molds. White empty arrowheads indicate mites with their digestive tracts filled with flour. Scale bar: 0.5 mm

Acarine parasites occur in most of the recognized acarine lineages, except for Opilioacarida (Krantz and Walter 2009; Walter and Proctor 2013). Most taxa among parasitic mites are parasites of vertebrates, invertebrates or plants (phytophages). However, at least *T. putrescentiae* displays an evident affinity to slime molds as well. Based on our observations, the interaction reported here appears closer to and stronger than what is called ‘insidious predation’ (Zelmer 1998) and seems much more than the food competition reported for *T. putrescentiae* (Xuan et al. 1993). Because live plasmodia and sclerotia were the least favorized stages of all appearing in the slime mold life cycle, it is disputable whether the mite is a parasite of slime molds. The relation should fulfil at least some criteria of the parasitism suggested by various authors, and/or at least some key features of this phenomenon should be depicted. It must be emphasized here that not all authors accept the full spectrum of the criteria listed below as typical of (and only of) parasitism. However, we can find among them the criteria fulfilled by the analyzed interaction:

1. The habitat relationship (Dogel’ 1966; Zelmer 1998; Weiner 2003). Judging by the ephemeral appearance of the mites in the slime mold cultures, the interaction revealed here is of a facultative or transient (periodic) nature, typical for an evolutionarily young relationship (Weiner 2003). What makes this relationship unique is that it is the microorganismal partner in this relationship, the slime mold, that is the host, and not the metazoan parasite, the mite.

2. The size relationship (Goff 1982; Weiner 2003). The maximal length and width of the body of a mature *T. putrescentiae* does not exceed 500–750 µm (Liu et al. 2006), whereas the size of a big plasmodium reared as a single individual may exceed hundreds of cm^2^ in area (Stephenson and Stempen 1994; Płonka and Rakoczy 1997; Adamatzky 2010). In our cultures, the area of a Petri dish (sometimes fully covered with the plasmodium) was ca. 60 cm^2^. In heavily infested cultures we found parts of the plasmodium ‘covered’ with mites side by side (Figs. 3a, g, 4b, 5d, Online Resources 1–3), and we estimated that the density of mites in such places could reach up to 250 per cm^2^, i.e., 20,000 mites per dish. It is comparable to the results by Smrž and Čatská (1987) who found up to 10,000 mites per dish of fungal cultures.

3. Immunity (Sprent 1962). The existence of naked plasmodia in the environment densely inhabited by microorganisms proves that the slime molds must possess some effective mechanisms of defense, like the evolutionarily ancient production of nitric oxide (Radomski et al. 1991; Płonka and Rakoczy 2000). Similarly, *F. septica* secretes fungicidal enzymes to the medium (Stephenson and Schnittler 2016). At present, we cannot identify any mechanism of the mycetozoal defense against the mites, but this may constitute a rationale for further research.

4. Evolution (decrease in host fitness, co-evolution, trophic selectivity and monophagy) (Crofton 1971a; Ewald 1995; Zelmer 1998; Weiner 2003). The overdispersed distribution of mites within the slime mold population, killing the heavily infected hosts (all stages of the slime mold life cycle, except spores), higher reproductive potential of the mite than the slime mold (the latter only exceptionally produces aethalia in vitro), and physiological dependence of the mite on the slime molds (only in mature cultures)—they all strongly point to a decrease in the host fitness in the cultures. However, some spores of *Didymium* sp. may germinate after passing through the digestive tract of a mite (Keller and Smith 1978), so the interaction sometimes bears the symptoms of protocooperation (facultative mutualism; Weiner 2003). A similar interaction between beetles and slime molds has been classified recently as mutualism (Sugiura et al. 2019). Similarly, mites of genus *Tyrophagus* sp. spread spores of ciliates, thus interacting positively, which was determined as mutualism and phoresy (Bharti et al. 2020).

5. The trophic relationship (Dogel’ 1966; Weiner 2003) or ‘dependence of the parasite on at least one gene of the host or on its product’ (MacInnis 1976; Zelmer 1998). This has been manifested strongly for at least several species of slime molds investigated here, and for different stages of their life cycle. We not only observed a quick disposal of a given type of slime mold material from the culture, but also a gradual filling of the digestive tract of the mites with the material of a particular coloration (Figs. 3, 4, 5, 6—white empty arrowheads)—white for the flour (Fig. 6), yellow for the plasmodium or the sclerotium (Figs. 3d, 5b), orange for the dead or decaying plasmodium (Fig. 3g), and brown/black for the spores (Figs. 3m, 4d, 5f; see also Keller and Smith 1978). The latter constitutes an evidence that the mites indeed feed on the mycetozoal material. Accordingly, the mites trophically depend on the slime mold, which not only provides the habitat, but also constitutes the source of food for the parasite.

It is a good opportunity to consider another type of relationship between the mite and the slime mold. In the in vitro cultures, oat flour is the main source of nutrients for the slime mold and for *T. putrescentiae* (Fig. 6, Online Resources 4), which may imply food competition (Xuan et al. 1993). In most cases *T. putrescentiae* has been regarded as synanthropic mite, whereas its occurrence in the soil has been commonly recorded (Smrž and Čatská 1987; Smrž et al. 2016). The major food tested for laboratory populations of this species have been stored products, yeasts or at least synanthropic fungi (Smrž and Čatská 1987; da Silvia et al. 2019; EPPO 2000). The food preference of *T. putrescentiae* towards fungal taxa indicates some specificity, but also plasticity in food requirements. The latter is also reflected in the array of vernacular names known for this mite (EPPO 2000).

Laboratory observations indicate that *T. putrescentiae* feeds on spores as well as on hyphae of a large range of dermatophytes, yeasts and molds, but does not feed on bacteria (Duek et al. 2001). The mites often migrate among cultures and thus may act as vectors. Hence, they can be considered as a potential pest of mycology stock cultures that may cause serious damage in microbiological laboratories (Duek et al. 2001). Sustaining of this mite on a variety of food with high protein and fat content, is likely to go beyond the feeding strategies typical of saprophagous organisms. *Tyrophagus putrescentiae* only partially reveals the characteristics of a true parasite, i.e., monophagy or a tendency towards trophic specialization (Weiner 2003). As a co-habitant of various species of slime molds, it cannot be called specific, nor is it found to co-evolve with its hosts. Importantly, to initiate a co-evolutionary relationship, a size relationship is necessary, often leading to trophic specialization of the co-evolving parasite (Weiner 2003).

Two more types of interactions should still be considered: parasitoidism and pseudo-parasitism. (1) With parasitoidism, the mite infestation can be a direct reason for the plasmodial death; however, the mites often feed on dead or decomposing plasmodia or sclerotia, thus becoming saprobionts or, better, necrophages (Weiner 2003). If feeding on live plasmodium or sclerotium really leads to necrosis of the material, the mite should be regarded here as a ‘facultative’ parasitoid (Croll 1973; Ewald 1995; Weiner 2003). Facultative, because transition from live to dead host body during mite feeding is not obligatory. (2) Pseudo-parasitism—defined as a transient, accidental presence and survival of the invading, normally free-living organism in the organism of the pseudo-host (Złotorzycka et al. 1998)—cannot be excluded, as the mites are free-living and lead a saprobiont-like life under natural conditions. However, in presence of the slime mold, the mites reveal strong affinity to the mycetozoan, and this situation should not be regarded as ‘accidental’ (Online Resources 1). It seems crucial to find out whether such a relation occurs also in the natural environment.

Judging by the time that elapsed between the publication by Keller and Smith (1978) and our observations (2013–2020), the appearance of *T. putrescentiae* in cultures of slime molds is not just a coincidence but an established affinity. It is evident that depending on the stage of life cycle of the slime mold, the type of interaction between the slime mold and the mite keeps changing. This is another feature of an evolutionarily young interaction (Ewald 1995; Weiner 2003), so it is particularly important to find examples of relationships between other mite species (and other organisms) and/or other types of slime molds. One should recall that *Tyrophagus* sp. enter also sophisticated and peculiar interactions with parasitic fungi (Xuan et al. 1993), and with ciliates (Bharti et al. 2020). The existing interactions may be a good starting point for further transformations, defining this group of mites as organisms of high evolutionary potential. Follow-up (mathematical) model studies (Crofton 1971a, b; Weiner 2003) will help paint a clear picture of such interactions in their various guises. The mycetozoan laboratory cultures in vitro, as vastly controllable, seem to constitute a particularly suitable system to explore such interactions.

## Conclusions

*Tyrophagus putrescentiae* and the slime molds *F. septica*, *P. polycephalum*, and *Didymium* sp. kept under in vitro conditions create an interesting system of mutual interactions bearing symptoms of several known ecological interactions (i.e., parasitism, pseudo-parasitism, parasitoidism, insidious predation, grazing, trophic competition, necrophagy, mutualism and phoresy) at various stages of their life cycle. Given the growing interest in slime molds, this plethora of relationships create a convenient model of study for further research, both under laboratory conditions and in the natural environment.

## Supplementary Information

Below is the link to the electronic supplementary material.

Supplementary file1 (MP4 209108 KB)

Supplementary file2 (MP4 301951 KB)

Supplementary file3 (MP4 184080 KB)

Supplementary file4 (MP4 143482 KB)

## Data Availability

The vouchers of the mite are deposited at the Department of Invertebrate Systematics and Ecology, Wrocław University of Environmental and Life Sciences, Poland. All the data and photo documentation are available from the authors on request.
